# Interplay of H2A deubiquitinase 2A-DUB/Mysm1 and the p19^ARF^/p53 axis in hematopoiesis, early T-cell development and tissue differentiation

**DOI:** 10.1038/cdd.2014.231

**Published:** 2015-01-23

**Authors:** M Gatzka, A Tasdogan, A Hainzl, G Allies, P Maity, C Wilms, M Wlaschek, K Scharffetter-Kochanek

**Affiliations:** 1 Department of Dermatology and Allergic Diseases, University of Ulm, Ulm 89091, Germany; 2 Institute of Immunology, University of Ulm, Ulm 89091, Germany

## Abstract

Monoubiquitination of core histone 2A (H2A-K119u) has a critical role in gene regulation in hematopoietic differentiation and other developmental processes. To explore the interplay of histone H2A deubiquitinase Myb-like SWIRM and MPN domain containing1 (2A-DUB/Mysm1) with the p53 axis in the sequential differentiation of mature lymphocytes from progenitors, we systematically analyzed hematopoiesis and early T-cell development using Mysm1^−/−^ and p53^−/−^Mysm1^−/−^ mice. Mysm1^−/−^ thymi were severely hypoplastic with <10% of wild-type cell numbers as a result of a reduction of early thymocyte progenitors in context with defective hematopoietic stem cells, a partial block at the double-negative (DN)1–DN2 transition and increased apoptosis of double-positive thymocytes. Increased rates of apoptosis were also detected in other tissues affected by Mysm1 deficiency, including the developing brain and the skin. By quantitative PCR and chromatin immunoprecipitation analyses, we identified *p19*^*ARF*^, an important regulator of p53 tumor suppressor protein levels, as a potential Mysm1 target gene. In newly generated p53^−/−^Mysm1^−/−^ double-deficient mice, anomalies of Mysm1^−/−^ mice including reduction of lymphoid-primed multipotent progenitors, reduced thymocyte numbers and viability, and interestingly defective B-cell development, growth retardation, neurological defects, skin atrophy, and tail malformation were almost completely restored as well, substantiating the involvement of the p53 pathway in the alterations caused by Mysm1 deficiency. In conclusion, this investigation uncovers a novel link between H2A deubiquitinase 2A-DUB/Mysm1 and suppression of p53-mediated apoptotic programs during early lymphoid development and other developmental processes.

According to the histone code model of transcriptional regulation, collaboration of histone modifying and chromatin remodeling enzymes with sequence-specific DNA binding transcription factors (TFs) in larger multiprotein complexes is required for the sequential differentiation of specialized cell populations from their progenitors, to activate and silence lineage-specific genes in a coordinated manner.^1, 2, 3, 4^ In this context, chromatin remodeling complexes of the SWI/SNF, ISWI and Mi-2/NuRD families control the accessibility of specific regions of DNA to lineage-specific TFs by regulating chromatin compaction, spacing of nucleosomes, histone–DNA contacts, and association with histone modifying enzymes.^5, 6, 7, 8, 9^ Moreover, aside from DNA methylation, covalent posttranslational modifications of the four core histones (H2A, H2B, H3, and H4) – including acetylation, methylation, phosphorylation, sumoylation, and ubiquitination – regulate target gene accessibility via introduction of permissive or repressive marks.^10, 11^

Components of the Polycomb Repressive Complex (PRC) 1, such as Bmi1, Mel18, and Ring1a/b, which recognizes trimethylated Histone H3 (3mH3K27) and competitively inhibits the SWI/SNF–chromatin remodeling complex, are established regulators of hematopoietic stem cell (HSC) maintenance and T-cell development.^12, 13^ In a heterodimetric complex with Bmi1, Ring1b possesses significant E3 ubiquitin ligase activity and mediates Polycomb silencing and X chromosome inactivation via histone H2A monoubiquitination at lysine 119 (H2A-K119u).^14, 15^ The ubiquitination status of histone H2A, a key histone in the DNA-damage response (DDR), is – other than by H2A ubiquitin ligases of the PRC1 – also controlled by non-PRC E3 ubiquitin ligases such as 2A-HUB/hRUL138 (Zhou *et al.*^16^), RNF8 (Huen *et al.*,^17^ Kolas *et al.*,^18^ and Mailand *et al.*^19^), and RNF168 (Doil *et al.*,^20^ Nicasso *et al.*,^22^ and Mattiroli *et al.*^21^), as well as by activating H2A deubiquitinases (H2A-DUBs), such as USP3 (Nicasso *et al.*,^22^), USP16/Ubp-M,^23, 24^ USP21, (Nakagawa *et al.*^25^), USP22 (Zhang *et al.*,^26^) and 2A-DUB,^27^ in context with co-activator complexes.^28, 29^ As per the current concept, H2A ubiquitination contributes to specific transcriptional repression programs, maintenance of genome stability, and DNA repair, whereas H2A deubiquitination is associated with transcriptional activation, cell cycle transition, as well as tuning of genome stability and target gene activation in cancer cells.^29, 30^

The histone H2A deubiquitinase 2A-DUB or Mysm1/Kiaa1915 (Myb-like SWIRM and MPN domain containing1) is a nuclear protein of 828 amino acids containing a SWIRM domain, a SANT (SWI-SNF, ADA N-CoR, TFIIIB) domain with DNA-binding activity and a JAMN/MPN domain with intrinsic metalloprotease-like activity.^27^ In prostate cancer cells, Mysm1 activates transcription of androgen receptor-regulated genes as part of a co-regulatory complex with histone acetyltransferase p300/CBP-associated factor by coordinating histone acetylation and deubiquitination, and by destabilizing the association of linker histone H1 with nucleosomes.^27^ More recently, analysis of Mysm1 knockout mice revealed additional functions of the H2A-DUB in regulation of hematopoietic development as Mysm1 deficiency resulted in – among other abnormalities – severe depletion of B cells and T cells, anemia, and thrombocytosis.^31, 32^ Comparable with the pathophysiology of mice lacking PRC1 component Bmi1,^33^ postnatal lymphopenia in Mysm1-deficient mice correlated with a depletion of HSC likely resulting from activation of the DDR and uncontrolled production of reactive oxygen species (ROS).^31^ In murine skin, Mysm1 deficiency was associated with atrophy (present article and own unpublished observations) and malformation of the tail.^34^

As it was until now unclear whether 2A-DUB/Mysm1 specifically regulates gene expression at a subset of gene promoters required for hematopoiesis or may have a broader impact on physiological processes in different tissues, we here performed a mechanistic investigation of Mysm1 function in the sequential differentiation of lymphocytes from HSCs and of its interactions with apoptotic pathways in general. To confirm the functional relevance of the interplay of 2A-DUB/Mysm1 and the p19^ARF^/p53 axis suggested by our gene expression and chromatin immunoprecipitation (ChIP) data, we generated p53^−/−^Mysm1^−/−^ double-knockout (DKO) mice. Defective lymphopoiesis and other developmental anomalies caused by Mysm1 deficiency were almost completely restored in p53^−/−^Mysm1^−/−^ mice revealing a novel interaction of the histone-modifying enzyme 2A-DUB/Mysm1 and the ARF/p53 pathway, with potential relevance for lymphoma and leukemia development and other diseases.

## Results

### 2A-DUB/Mysm1 regulates critical checkpoints of early lymphopoiesis and thymocyte development

**Figure 1:**
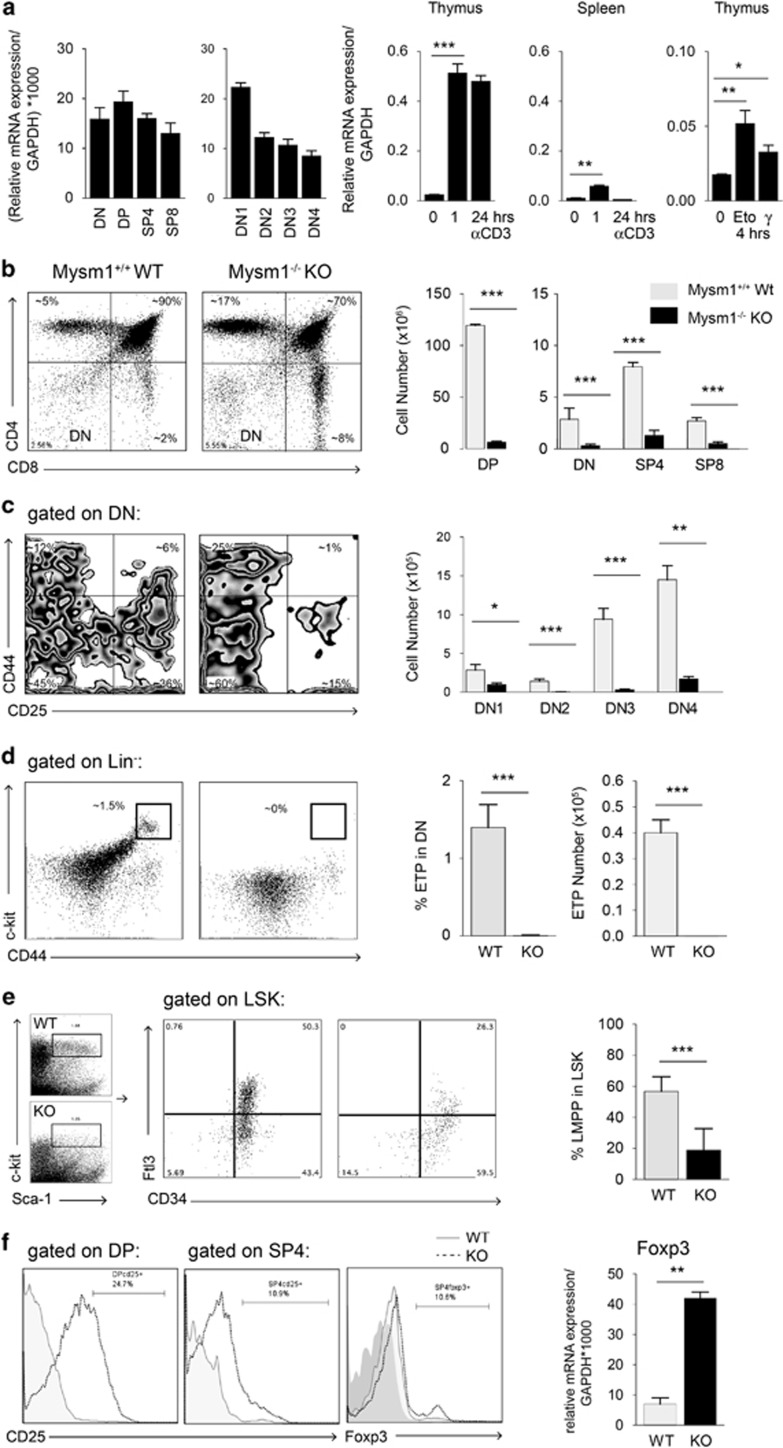
Thymocyte development and lymphopoiesis are impaired at critical checkpoints in the absence of 2A-DUB/Mysm1. (**a)** Mysm1 mRNA expression of indicated thymocyte subsets FACS sorted from 4- to 6-week-old C57BL/6 mice relative to GAPDH (left two bar graphs). Induction of Mysm1 mRNA in thymocytes and splenocytes stimulated with αCD3 (1 *μ*g/ml) for indicated time periods and thymocytes 4 h after exposure to etoposide (eto, 10 *μ*M) or *γ*-irraditation (*γ*, 4 Gy) relative to GAPDH (right bar graphs). In all qPCR analyses, bar graphs represent mean expression of a least three mice and two individual experiments ±S.D. (**b**–**e)** Flow cytometric analysis of total (CD19, Mac1, Gr1, Ter119, NK1.1, and Epcam1-negative) or lineage (CD4, CD8, CD3, CD19, Mac1, Gr1, Ter119, NK1.1, and *γδ*TCR)-negative (Lin_neg_) thymocytes or Lin_neg_ BM cells from 4- to 6-week-old male Mysm1^−/−^ (KO) mice in comparison with age-matched WT littermates (dot plots from representative experiments are shown, *n*>8). Total cell numbers (shown in bar graphs next to the FACS profiles) of indicated subsets represent averages from at least six mice and three independent experiments ±S.D. (**f)** Dot plots and histograms of CD25 and Foxp3 expression of indicated thymocyte subsets and correlation with Foxp3 mRNA expression of total Mysm1^+/+^ (WT) or Mysm1^−/−^ (KO) thymocytes. FACS data show representative examples of at least three separate experiments with two WT (grey lines) and two KO (grey dotted lines) thymi relative to isotype control Ab (grey area). Bar graphs show means of three experiments ±S.D.

In a quantitative PCR (qPCR) screening approach, we identified *2A-DUB/Mysm1* as a gene expressed throughout the double-negative (DN) 1–4 populations in the thymus, in double-positive (DP), single-positive (SP)4, and SP8 thymocytes, and regulated by stimulation with T-cell receptor (TCR) agonists, such as anti-CD3, in thymocytes and peripheral T cells *in vitro*, as well as by inducers of apoptosis and DNA damage such as etoposide and *γ*-irradiation, in thymocytes (Figure 1a). Among the anomalies of Mysm1^−/−^ mice, especially defects in the bone marrow (BM) – resulting in lymphopenia, anemia, and thrombocytosis – and in lymphocytes have been described.^31, 32^ However, no conclusive data are available so far on the function of Mysm1 in the thymus or in other tissues. To further evaluate the causes of defective lymphocyte development and pinpoint stage-specific alterations, we first systematically assessed Mysm1^−/−^ BM and thymi. Thymi from 4-week-old Mysm1-deficient mice were severely hypoplastic with an average total cellularity of <10% of wild-type thymi, resulting in a decrease of the absolute T-cell number ratio in Mysm1^−/−^ *versus* Mysm1^+/+^ secondary lymphoid organs by up to factor 10 (Supplementary Figure 1a). Whereas a consistent decrease in Mysm1^−/−^ DN, DP and SP thymocyte numbers was detectable, analysis of thymocyte subset distribution revealed a variable relative increase of SP and DN at the expense of DP thymocytes (Figure 1b). Within the DN stages, the most severe reduction in total numbers was consistently detectable in the DN2 subset (average ~55-fold reduction) and the DN3 subset (average ~25-fold reduction), whereas DN1 and DN4 fractions were relatively increased – indicating a partial block at the DN1–DN2/3 transition in Mysm1^−/−^ thymi (Figure 1c). In addition, the most primitive CD44^+^CD25^−^c-kit^high^ thymocyte precursors, called early thymocyte progenitors (ETPs), which still possess some multipotentiality and usually account for up to 3% of Lin^neg^ thymocytes,^35, 36, 37^ were almost undetectable in 4-week-old Mysm1^−/−^ thymi by FACS analysis (Figure 1d).

The lack of ETPs correlated with significantly reduced total BM cellularity in young Mysm1^−/−^ mice, in particular with significant relative decreases of the CD34^+^Flt3^+^ lymphoid-primed multipotent progenitor (LMPP) fraction within the Lin^neg^Sca-1^+^c-kit^high^ (LSK) population to an average of 20% compared with 55% in wild-type littermates (Figure 1e). In BM reconstitution experiments, Mysm1^−/−^ Lin^neg^ BM progenitor cells were severely impaired in their ability to reconstitute T-cell and B-cell lineage in lethally irradiated Rag2-deficient recipients in comparison with wild-type cells and to give rise to longer-term multi-lineage engraftment confirming intrinsic defects of Mysm1-deficient hematopoietic cells (Supplementary Figure 1b). These data strongly suggest that defective HSC maintenance and differentiation contribute to defective lymphoid development in 2A-DUB/Mysm1-deficient mice. In ontogeny, defective thymic development was already detectable in newborn Mysm1^−/−^ mice on day 2 after birth, presenting with significantly smaller thymi and an up to fivefold reduction in total thymocyte numbers compared with wild-type littermates (Supplementary Figure 1c). Detailed analysis of the DP and SP subsets in Mysm1^−/−^ thymi consistently revealed relative increases in the fraction of CD25^+^ DP and SP4 thymocytes (Figure 1f, left). Increased CD25/IL-2Rα levels of developing Mysm1^−/−^ thymocytes correlated with upregulation of Foxp3^+^ protein levels and mRNA expression as detected by FACS analysis and qPCR (Figure 1f, right), and may confer a survival advantage during selection.

### Increased apoptosis is a common denominator of Mysm1-deficient BM progenitors, thymi, and other affected tissues

**Figure 2:**
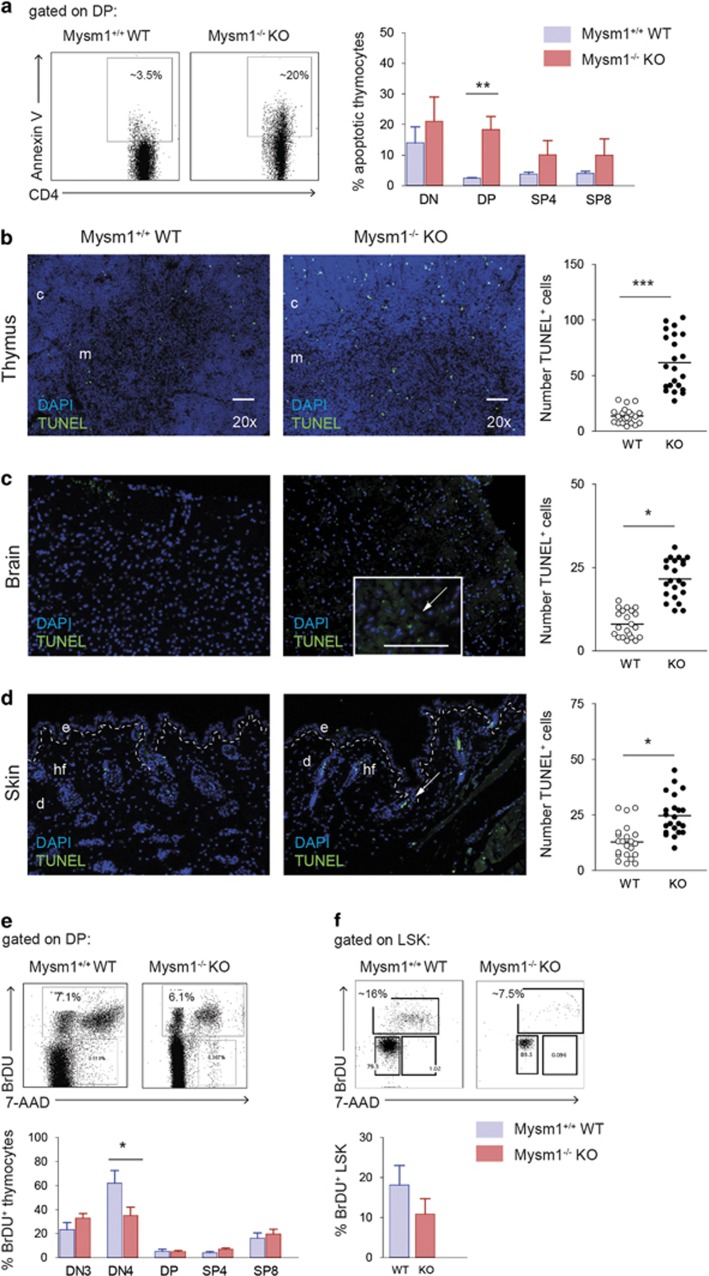
Increased apoptosis is a common denominator of Mysm1-deficient BM progenitors, thymi, and other affected tissues. (**a**) Significantly increased Annexin V-positive apoptotic cell fractions in Mysm1^−/−^ KO thymi in FACS analyses (representative FACS plots shown). Bar graphs represent mean values of six to eight age-matched mice of each genotype. (**b**) TUNEL assays performed on frozen tissue sections, to detect apoptosis in Mysm1^−/−^ (KO) thymi *in situ* compared with age-matched wild-type (WT) controls (nuclei blue, TUNEL green, white bar corresponds to 10 *μ*m; *n*=4, representative photographs shown, original magnification as indicated). To quantify TUNEL^+^ apoptotic cells, positively stained cells were counted in 12–15 high-power fields (HPFs), and distributions and medians presented in scatter plots for all tissues analyzed (*n*≥3). (**c**) Detection and quantification of TUNEL-positive cells (green) in Mysm1^−/−^ brain (neocortex) and (**d**) skin sections compared with controls (*n*=3, representative stainings shown; e, epidermis; d, dermis; hf, hair follicle; white arrows point to apoptotic cells). (**e**) Overall proportions of BrdU incorporating LSK cells from BM prepared 2 h after BrdU pulse (representative plots shown). (**f**) Proportions of BrdU incorporating thymocytes of indicated subsets prepared 2 h after a BrdU pulse measured by FACS analysis (representative plots shown, *n*>3, bar graphs represent average values of at least three mice of each genotype)

To explore the mechanisms of the developmental defects caused by Mysm1 deficiency, we subsequently systematically analyzed apoptosis and proliferation during lymphopoiesis, and in tissues affected by Mysm1 deficiency. In Mysm1^−/−^ thymi, the fraction of apoptotic cells was most significantly increased within the DP subset accounting for up to 30% of DP Mysm1^−/−^ thymocytes compared with ~3–5% in wild-type controls as measured by Annexin V/PI staining (Figure 2a). Increased apoptosis of Mysm1^−/−^ DP thymocytes may therefore, at least in part, contribute to the reduction in thymocyte numbers. In Mysm1^−/−^ BM, we observed overall increased apoptosis within the LSK population consistent with previous reports.^31^

Increased thymocyte apoptosis in Mysm1-deficient mice was confirmed *in situ* by terminal deoxynucleotidyltransferase dUTP nick end labeling (TUNEL) staining of frozen tissue sections from 4-week-old Mysm1-deficient mice compared with their wild-type littermates (Figure 2b). Mysm1^−/−^ thymi contained high numbers of TUNEL-positive apoptotic cells, especially at the cortico-medullary junction, whereas only few TUNEL^+^ cells were present in wild-type sections. Similarly, the number of *γ*H2 AX foci was significantly increased in Mysm1^−/−^ thymi indicative of increased DNA damage (Supplementary Figure 2a). Increased apoptotic fractions correlated with increases in ROS in Mysm1-deficient thymocytes as detected by 2′-7′-dichlorodihydrofluorescein diacetate staining in FACS analysis (Supplementary Figure 2b). Increased fractions of TUNEL^+^ cells were also observed in Mysm1^−/−^ brain (Figure 2c) and the skin (Figure 2d), pointing to a common mechanism in affected tissues.

In 4-week-old Mysm1^−/−^ mice, no consistent increase of cycling Mysm1^−/−^ HSCs in the steady state was detectable compared with controls after a 2-h bromodesoxyuridine (BrdU) pulse. Instead, the fraction Mysm1^−/−^ LSK cells in S-phase measured by BrdU and 7-AAD staining was reduced or normal (Figure 2e). Analysis of BrdU incorporation of different thymocyte subsets after a 2-h pulse revealed significantly decreased proliferation of Mysm1^−/−^ DN4 thymocytes, whereas fractions cycling BrdU^+^ cells within the other thymocyte subsets were normal or slightly increased compared with controls (Figure 2f). Despite reduced DN3 cell numbers and reduced proliferation of DN4 cells, upregulation of the TCRβ chain was not defective (Supplementary Figure 2c). In addition, slightly increased homeostatic proliferation of Mysm1^−/−^ peripheral T cells was detectable (Supplementary Figure 2d), and proliferation of purified Mysm1^−/−^ peripheral T cells in response to plate-bound αCD3 as measured by simultaneous carboxyfluorescein succinimidyl ester and Annexin V staining of CD4^+^ and CD8^+^ T cells on day 4 was comparable to control T cells (Supplementary Figure 2e). To investigate the response of Mysm1^−/−^ thymocytes to different inducers of apoptosis, thymocyte apoptosis assays were performed measuring Annexin V-positive cells after a 24-h culture period revealing a slightly increased susceptibility of Mysm1^−/−^ DP thymocytes to DNA-damage induction by etoposide and *γ*-irradiation compared with wild-type counterparts (Supplementary Figures 3a and b).

### Increased apoptosis correlated with upregulation of p19^ARF^ and induction of p53 target genes in Mysm1-deficient thymocytes and the BM

**Figure 3:**
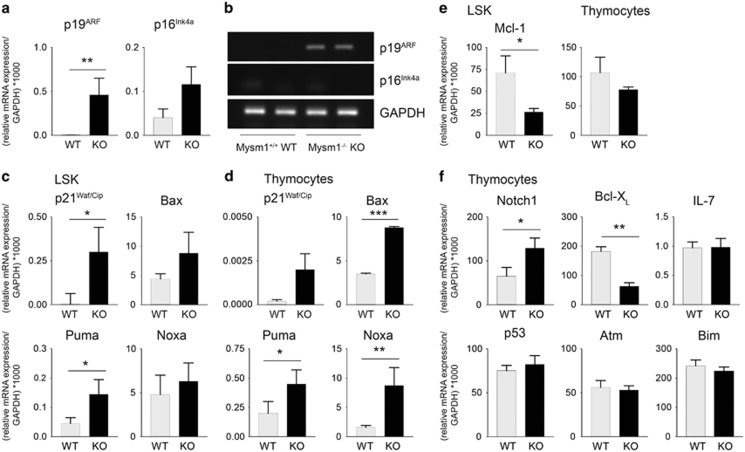
Increased apoptosis correlated with induction of pro-apoptotic 19^ARF^ mRNA and of p53 target genes in Mysm1-deficient BM and thymi. (**a**) qPCR was performed with primers specific for p19^ARF^ or p16^Ink4a^ using cDNA from total thymocytes of Mysm1^−/−^ (KO) or controls (wild type (WT)). Significantly increased p19^ARF^ mRNA level in Mysm1^−/−^ compared with WT thymocytes relative to GAPDH mRNA detected by qPCR. Results represent mean fold changes of three independent experiments, with two thymi of each genotype. (**b**) Gel electrophoresis of representative Mysm1^−/−^ and WT PCR samples. (**c**) qPCR detection of p53 target genes *p21*^*Waf/Cip*^, *Bax*, *Puma*, and *Noxa* in LSK cells from Mysm1^−/−^ BM and (**d**) in total Mysm1^−/−^ thymocytes compared with controls relative to GAPDH. (**e**) Decreased relative Mcl-1 mRNA expression in Mysm1^−/−^ BM. (**f**) qPCR performed with primers specific for indicated genes on Mysm1^−/−^ (KO) or control (WT) cDNA from total thymocytes with *GAPDH* as the reference gene. Bar graphs show mean expression values ±S.D. of at least three independent experiments

To investigate the mechanisms underlying the striking increase in BM and thymocyte apoptosis on Mysm1 deletion, expression of pro- and anti-apoptotic genes, and cytokine genes known to be important regulators of blood cell survival and development were analyzed on the mRNA level by qPCR. Of note, among the pro-apoptotic genes of interest, in particular highly significant increases in p19^ARF^ mRNA of up to 100-fold were detectable in total Mysm1-deficient thymocytes from 4-week-old mice compared with their wild-type controls (Figure 3a, right). Using specific primer sets for p19^ARF^ and p16^INK4a^ mRNA, we excluded that the detected increase resulted from general induction of the Cdkn2a locus. In contrast to p19^ARF^, p16^INK4a^ mRNA was only slightly induced in Mysm1-deficient thymocytes (Figure 3a (left) and Figure 3b). In order to confirm p19^ARF^ upregulation on the protein level, immunofluorescent (IF) analyses of frozen sections of 4-week-old Mysm1-deficient thymi were then performed with an antibody against p19^ARF^ (Supplementary Figure 3c). In addition, we found increases in p53, the major target of p19^ARF^, in the cortex and the medulla of Mysm1-deficient thymi and in Mysm1-deficient skin (Supplementary Figures 3d and e). Defects of Mysm1^−/−^ LSK cells in the BM and of Mysm1^−/−^ thymocytes correlated with significant upregulation of p53 target genes such as *Bax* and *p21*^*Waf/Cip*^, as well as *Puma* and *Noxa* (Figures 3c and d). Among other survival genes differentially expressed in Mysm1^−/−^ and wild-type hematopoiesis, Mcl-1 mRNA levels were significantly decreased by factor 3 in Mysm1^−/−^ BM (Figure 3e), whereas decreases in Bcl-X_L_ mRNA were detectable to the same extent in Mysm1^−/−^ thymi (Figure 3f). In addition, we found slightly increased expression of Notch1 mRNA. As no prominent alterations in IL-7, IL-7R, Bim, Atm, Gfi-1, ROR*γ*t or Bmi1 mRNA expression were detectable (Figure 3f and not shown), we speculated that deregulated lymphoid development and increased apoptosis in Mysm1^−/−^ mice could be mediated at least in part by the p19^ARF^/p53 axis.

### Rescue of growth defects and lymphoid progenitor differentiation in p53^−/−^Mysm1^−/−^ DKO mice

**Figure 4:**
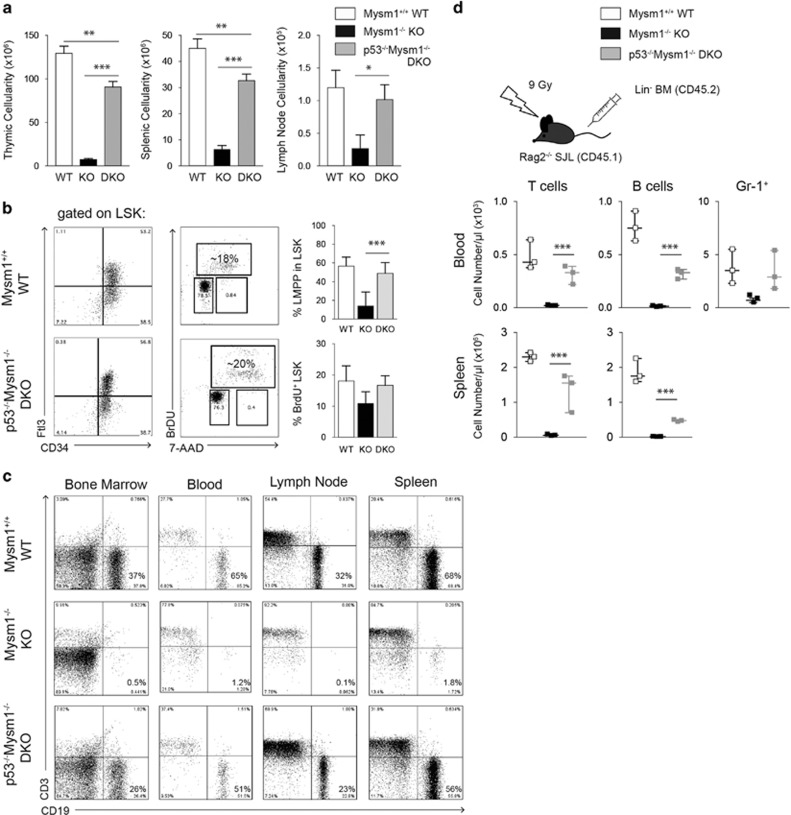
Partial restoration of LMPPs, B-cell development, and peripheral B-cell numbers in p53^−/−^Mysm1^−/−^ DKO mice. (**a**) Thymic, splenic, and LN total cellularities in 4-week-old p53^−/−^Mysm1^−/−^ (DKO) mice in comparison with Mysm1^−/−^ (KO) and wild-type (WT) littermates. Data represent mean values of at least four mice of each genotype ±S.D. (**b**) FACS analysis of BM cells of p53^−/−^Mysm1^−/−^ (DKO) and WT mice for Lin^−^Sca-1^+^c-kit^high^ (LSK) cells and CD34^+^Flt3^+^ (LMPP) cells within the LSK population (gating strategy as indicated, representative plots of at least three independent experiments shown). (**c**) FACS analysis of single-cell suspensions prepared from the BM, peripheral blood, spleens, and the LNs from p53^−/−^Mysm1^−/−^ (DKO) and WT mice with anti-CD3 and anti-CD19 to discriminate T and B cells, respectively. Representative plots from three independent experiments are shown. (**d**) Reconstitution potential of p53^−/−^Mysm1^−/−^ Lin^−^ BM cells was measured on week 8 after transfer into lethally irradiated Rag2^−/−^ recipients by analysis of peripheral blood and spleens for the presence of T cells and B cells. Scatter plots show cell counts of individual recipient mice and medians of two separate experiments

In order to validate the potential role of p53 as mediator of elevated apoptosis and defective lymphopoiesis in Mysm1^−/−^ mice, we generated DKO mice deficient for both Mysm1 and p53 (p53^−/−^Mysm1^−/−^) by crossing Mysm1-deficient mice (Mysm1^tm1a/tm1a/Komp/Wtsi^) onto the p53-deficient background. p53^−/−^Mysm1^−/−^ DKO mice were born roughly at Mendelian ratios, but with clear preference for male offspring. Consistently, significantly increased death rates of female p53^−/−^ embryos had been observed caused primarily by defects in neural tube closure.^38, 39^ Strikingly, the visual phenotypic anomalies of Mysm1-deficient mice were largely abolished on the p53-null background, and p53^−/−^Mysm1^−/−^ DKO mice appeared normal in size, weight, and coat color compared with C57BL6/J wild-type littermates (Supplementary Figure 4a). Mysm1^−/−^ mice homozygous or heterozygous for the p53 deletion (null-allele) were subsequently used to analyze the role of p53 in BM and immune cells. Absence of functional mRNA for both gene products, 2A-DUB/Mysm1 and p53, was confirmed in the BM, thymocytes, and the splenocytes of all DKO mice used for phenotypic analysis by qPCR (Supplementary Figure 4b). Thymi and spleens of 4-week-old p53^−/−^Mysm1^−/−^ DKO mice were roughly of normal size. Total cellularities of p53^−/−^Mysm1^−/−^ BM, thymi, spleens, and lymph nodes (LNs) were only slightly reduced, and on average at least 80% of age-matched wild-type controls, indicating a significant rescue of lymphopoiesis in DKO mice (Figure 4a). In p53^+/−^Mysm1^−/−^ (p53het) mice, a slight but variable recovery of BM, thymic, and splenic cell numbers could be observed compared with Mysm1^−/−^ mice (not shown).

As cellularities of BM and lymphoid organs were partially restored in p53^−/−^Mysm1^−/−^ DKO mice, we subsequently examined the presence of stem cell populations and lymphoid progenitors in the BM and of mature B cells in peripheral lymphoid organs of 4-week-old DKO mice and age-matched littermates. Strikingly, in the p53^−/−^Mysm1^−/−^ BM, HSC differentiation and LMPPs were restored (Figure 4b, left panel). BrdU incorporation assays indicated that proliferation of the LSK population in DKO mice was similar to wild-type HSC (Figure 4b, right panel). In addition, B-cell progenitors were recovered in the BM of young p53^−/−^Mysm1^−/−^ mice to around 80% of wild-type levels as indicated by the distribution of CD19-positive B lineage (on average 27.5% in DKO compared with 35% in wild type) (Figure 4c, first panel). Similarly, the CD19^+^ B-cell fraction in the blood, spleens, and the LNs of DKO mice was only slightly reduced compared with wild-type littermates (Figure 4c, right panels). In BM reconstitution experiments, p53^−/−^Mysm1^−/−^ Lin^−^ BM cells reconstituted T cells, and to a lesser extent also B cells, in lethally irradiated Rag2-deficient recipients at significantly greater rates than Mysm1^−/−^ donor cells, indicating that the intrinsic defect of Mysm1^−/−^ hematopoietic progenitors was partially restored on p53 ablation (Figure 4d). At the gene expression level, p53 ablation correlated with restoration of increased expression of p21^Waf/Cip1^ as well as Bax, Puma, and Noxa mRNA in p53^−/−^Mysm1^−/−^ BM and thymocytes, respectively, and interestingly, also decreased Mcl-1 mRNA levels were partially restored (Supplementary Figure 4c).

### Partial rescue of defective thymocyte development in p53^−/−^Mysm1^−/−^ DKO mice

**Figure 5:**
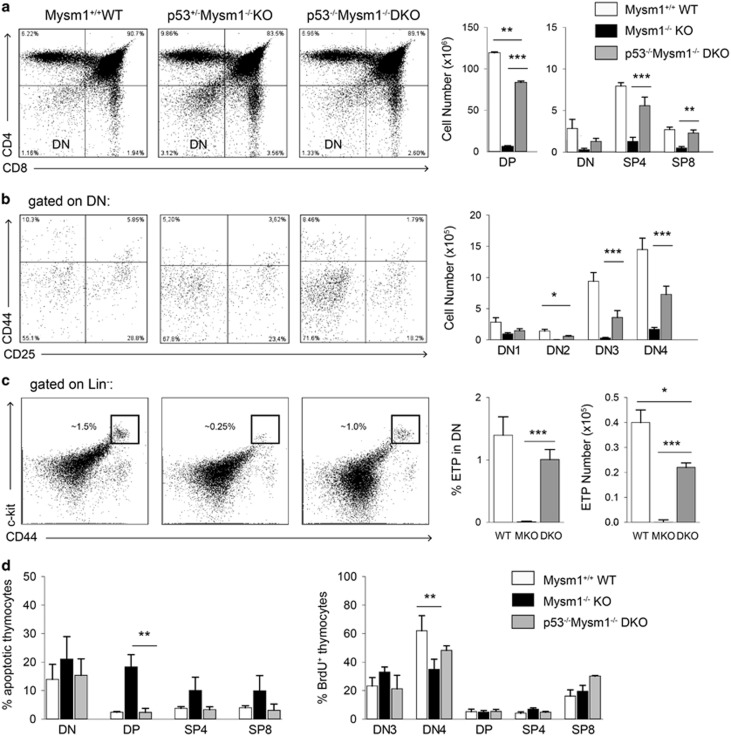
Partial rescue of defective T-cell development by p53 deletion in p53^−/−^Mysm1^−/−^ mice. (**a**–**c**) FACS analysis of thymocyte subpopulations of 4-week-old Mysm1^+/+^ (WT), p53^het^Mysm1^−/−^ and p53^−/−^Mysm1^−/−^ (DKO) mice (representative dot plots of three independent experiments shown). Total cell numbers of indicated thymocyte subsets represent mean values of at least four mice of each genotype (bar graphs). (**d**) Bar graphs indicate percentages of apoptotic rates in indicated DKO, KO and WT thymocyte subsets by Annexin V staining (left panel) and BrdU-positive cell fractions after a 2-h pulse (right panel). Data represent mean values of at least three independent experiments

FACS analyses of p53^−/−^Mysm1^−/−^ DKO thymi revealed roughly normal distribution of DN, DP, SP4, and SP8 thymocyte subpopulations in comparison with wild-type thymi (Figure 5a). However, the distribution of DN1-4 subsets in DKO thymi still indicated a subtle partial block at the DN1–DN2/DN3 and a concomitant moderate reduction of the cell number of DN2 thymocytes – comparable to Mysm1^−/−^ thymi – as residual phenotype (Figure 5b) pointing to a potential p53-independent regulatory mechanism at this stage. Remarkably, consistent detection of the Lin^−^CD44^+^CD25^−^c-kit^high^ fraction in DKO thymi indicated that the ETP was restored on p53 deletion to on average almost 85–90% of wild-type levels (Figure 5c). Partial restoration of thymic development could already be observed in most p53^+/−^Mysm1^−/−^ mice, suggesting a gene–dose effect (Figures 5a–c). Analysis of thymocyte apoptosis by Annexin V staining showed a restoration of DN and DP thymocyte viability, and apoptotic rates of p53^−/−^Mysm1^−/−^ thymocytes were similar to wild-type thymocytes (Figure 5d, left). Moreover, proliferation of p53^−/−^Mysm1^−/−^ thymocytes measured by BrdU incorporation resembled the wild-type pattern (Figure 5d, right).

### 2A-DUB/Mysm1 binds to a fragment of the *p19^ARF^* promoter in activated wild-type thymocytes

**Figure 6:**
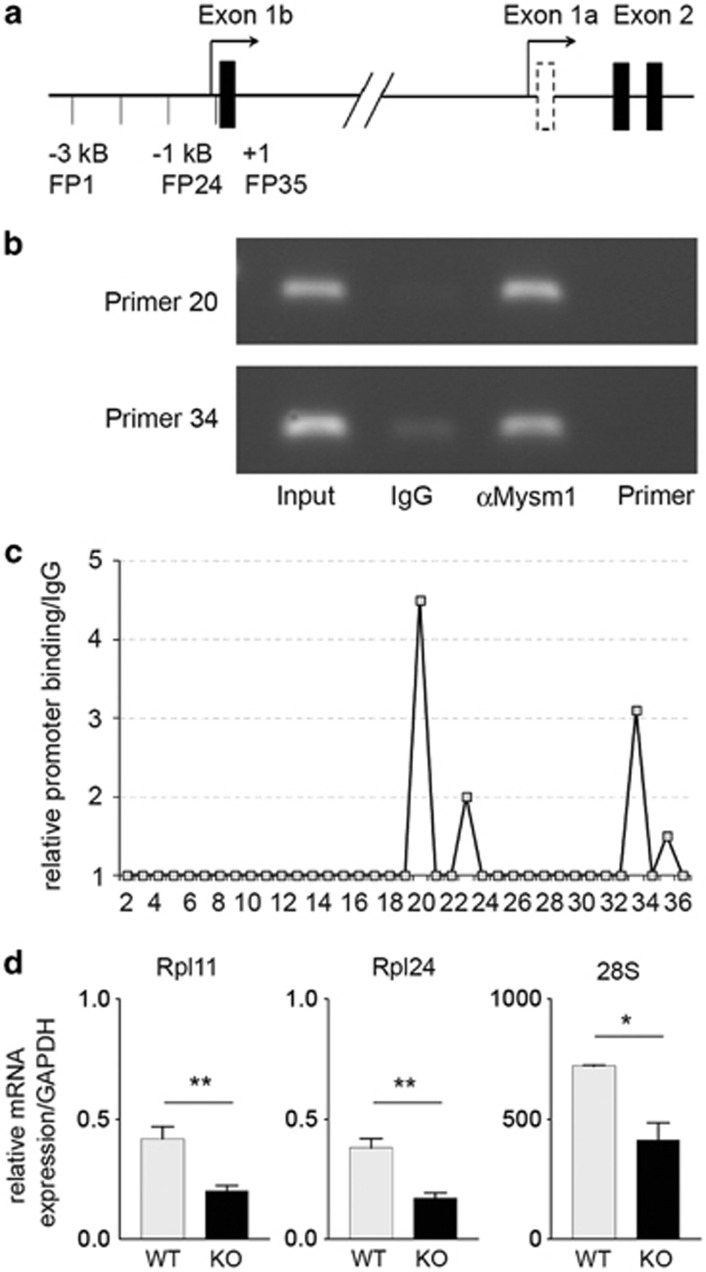
2A-DUB/Mysm1 binds to the *Cdkn2a (Ink4a/Arf)* promoter region. (**a**) Graphical analysis of the murine *Cdkn2a* promoter region and location of primer sets for the ChIP experiments. (**b**) Total thymocytes of 4-week-old C57BL/6 mice were stimulated with α-CD3 (1 *μ*g/ml) for 2 h and used for ChIP assays with an antibody against Mysm1 followed by PCR or qPCR, with primers for each amplicon of the *p19^ARF^* promoter (as indicated in **a**). (**c**) Normalized intensity of PCR results from two independent experiments as described above. Probes with an intensity increased in α-Mysm1 ChIP of threefold or more compared with IgG controls were defined as positive. (**d**) mRNA expression of ribosomal proteins Rpl11 and Rpl24, and of ribosomal S28 RNA in wild-type and Mysm1^−/−^ BM LSK cells relative to GAPDH (bar graphs represent mean values from at least three mice of each genotype and two independent experiments with S.D.)

As increased p19^ARF^/p53 activation in developing Mysm1-deficient mice could be a consequence of checkpoint activation, a default pathway, or be induced as a result of a lack of 2A-DUB/Mysm1 action in regulatory complexes involved in repression of the *Cdkn2a (Ink4/Arf)* locus, we next set out to explore Mysm1 binding to fragments of the *p19^ARF^* promoter in activated thymocytes. In order to screen for Mysm1 binding sites at the *p19^ARF^* promoter, a 3-kb sequence upstream of the *Cdkn2a* locus (−3000) spanning the transcriptional start site and exon 1B, was analyzed by ChIP with overlapping primer sets^1, 2, 3, 4, 5, 6, 7, 8, 9, 10, 11, 12, 13, 14, 15, 16, 17, 18, 19, 20, 21, 22, 23, 24, 25, 26, 27, 28, 29, 30, 31, 32, 33, 34, 35, 36, 37^ in semiquantitative and qPCR (illustrated in Figure 6a). Significant Mysm1 binding was detectable to a fragment −1-k upstream of the transcriptional start site (primer set 20) and to a fragment around the transcriptional start site of exon 1B (primer set 34) in activated thymocytes (Figure 6b). In comparison with IgG-negative controls, amplification was increased between 3.1- (primer set 34) and 4.5-fold (primer set 20) (Figure 6c). In conclusion, these results indicate that 2A-DUB/Mysm1 has the potential to bind to the *p19^ARF^* promoter and that enforced p19^ARF^ repression may be required to prevent apoptotic programs in activated thymocytes. In addition, in Mysm1^−/−^ BM progenitors, significant reduction in ribosomal RNA and large ribosomal proteins (Rpl) 11 and Rpl24 could be detected by qPCR, potentially accounting for an alternative mechanism of p53 activation (Figure 6d).

## Discussion

The major finding of the present investigation is that histone H2A deubiquitinase 2A-DUB/Mysm1 is critically required in lymphoid progenitors, developing thymocytes, and other cell types for protection from p53-mediated apoptotic programs. As defective lymphopoiesis of Mysm1-deficient mice could be rescued almost completely by deletion of p53, and also other anomalies – including growth retardation, neurological deficit, and skin atrophy – were ameliorated in p53^−/−^Mysm1^−/−^ DKO mice, interplay of Mysm1 and p53 appears to be a common principle in several sequential differentiation processes. In our systematic analysis of hematopoiesis in Mysm1^−/−^ mice, we identified a first block at the stage of HSCs and the transition to LMPPs. In line with previous studies, HSC were numerically reduced ^31, 32^ and cycling of LSK cells was impaired – both affecting early lymphoid development. Mechanistically, activation of p53-dependent pathways was responsible for these alterations in Mysm1^−/−^ HSC, because HSC numbers, cycling, and differentiation were rescued on p53 deletion in young mice. As LMPPs give rise to T-cell and B-cell precursors,^40^ and B-cell development was largely restored in p53^−/−^Mysm1^−/−^ DKO mice, B-cell depletion in the absence of Mysm1 is likely caused at least in part by a p53-mediated loss of progenitors. Consistent with a progenitor defect, we also demonstrated here that the ETP population was almost completely absent in Mysm1^−/−^ thymi. As additional alterations in subsequent phases of Mysm1^−/−^ thymic development could be identified, such as a partial block at the DN1–DN2/D3 stage and increased DP apoptosis, Mysm1 most likely fulfills intrinsic functions in the thymocytes, which need to be confirmed in additional studies using conditional lymphoid- or T-cell-specific deletion of Mysm1.

The requirement of 2A-DUB/Mysm1 for protection from cell death during hematopoiesis, thymic T-cell development, and in certain tissues shows a number of parallels to functions of other H2A-modifying epigenetic regulators, in particular Polycomb group (*PcG*) genes. PRC1 components Bmi1, Mel18, and Phc1/Rae28 modulate the self-renewal capacity of HSC in part by interaction with the *Cdkn2a (Ink4a/Arf)* locus.^41, 42, 43, 44^ Importantly, Bmi1 is not only required for maintenance of adult self-renewing HSC and their protection from ROS-induced DNA damage, but also regulates thymocyte development.^33, 45^ At the DN3/4 transition, Bmi1 is essential for survival of pre-T cells by suppressing p19^ARF^ expression during β-selection.^46^ Interestingly, deletion of p53 partially restored the defects of Bmi1^−/−^ thymocytes including the CD4 and CD8 profiles,^47^ very similar to the effect of p53 deletion in Mysm1-deficient lymphopoiesis. Moreover, provision of antioxidant *N*-acetylcysteine improved hematopoietic defects and counteracted impaired mitochondrial function, increased ROS levels, and engagement of the DDR pathway caused by absence of Bmi1.^33^

In our systematic analysis of apoptotic mediators, we found that DNA damage marker *γ*H2AX, p19^ARF^, and p53 levels, as well as ROS were increased in Mysm1-deficient thymocytes and, in part, in other affected tissues as well. In mRNA expression analyses, induction of p19^ARF^, the major regulator of p53, was the most prominent change in Mysm1^−/−^ compared with wild-type thymocytes. In addition, we demonstrated that 2A-DUB/Mysm1 has the potential to directly bind to the *Cdkn2a/p19^ARF^* promoter. These data suggest that p53-mediated pathways could – at least in thymocytes – be triggered through a direct effect of Mysm1 on p19^ARF^ expression. As PRC components, such as Bmi1, have been implicated in suppression of p19^ARF^ expression,^46^ it is possible that Mysm1 acts as part of a bigger complex regulating sequential deubiquitination and ubiquitination of the *p19^ARF^* promoter during different phases of thymocyte development. Consistently, a dynamic balance of histone H2A ubiquitination and deubiquitination was also suggested by earlier experiments in *Drosophila*, showing that Polycomb repressive DUB-mediated H2A deubiquitination is required for gene repression by PRC1.^48^

In Mysm1^−/−^ BM progenitors, reduction of ribosomal proteins Rpl11 and Rpl24 could contribute to the activation of p53-dependent pathways and, interestingly, belly spot and tail (Bst) mice, carrying a hypomorphic *Rpl24* gene mutation, display a similar p53-dependent skin phenotype and other anomalies comparable to Mysm1^−/−^ mice.^49^ Alternatively, checkpoint activation in Mysm1^−/−^ HSC and DP thymocytes could be initiated by master kinase ATM – which responds to double-strand break (DSB) DNA damage and disruptions in chromatin structure,^50, 51^ as well as to accumulation in intracellular ROS levels,^52, 53^ and can induce apoptosis via downstream target p53.^54^ During negative selection, TCR-induced apoptosis of DP and SP thymocytes could moreover be mediated via the p19^ARF^/p53 pathway downstream of E2F1.^55,56^ Although we did not observe upregulation of ATM or E2F1 mRNA in Mysm1-deficient thymi or BM in our gene expression analysis, increased phosphorylation or activity of ATM and E2F1 may occur in Mysm1-deficient cells as a result of increased ROS levels. Mechanistically, ROS, occurring as byproducts of normal cellular respiration and as defence mechanism in the immune system, could act either upstream as a trigger factor of p53 via induction of DNA DSB or as its downstream mediators of apoptosis.^57^

Potential p53 target genes that could mediate the defects observed in Mysm1^−/−^ HSC, lymphocytes, and other cell types include – apart from p21^Waf/Cip1^ and Bax – in particular BH3-only genes *Puma* and *Noxa*, both associated with hematopoietic cell death in response to stress such as cytokine deprivation, ionizing irradiation, and cytotoxic agents.^58, 59^ Although survival of DP thymocytes and HSC was restored in p53^−/−^Mysm1^−/−^ mice, overall thymic cellularity and B-cell recovery was still on average only 80% of wild-type mice pointing to contributions of p53-independent mechanisms. Consistently, apart from upregulation of p19^ARF^ mRNA, levels of anti-apoptotic Mcl-1, critical for survival of hematopoietic progenitors,^60^ and Bcl-X_L_ mRNA were significantly reduced in Mysm1^−/−^ BM and thymi, respectively, whereas mRNA expression of TFs ROR*γ*t, Id2, and Egr3, all regulators of Bcl-X_L_ in thymocytes,^61, 62, 63^ as well as of pro-apoptotic regulator Bim,^64^ was not altered.

To fully understand the function of 2A-DUB/Mysm1 in development and disease processes, more detailed characterization of the interplay of Mysm1 and p53 at the molecular level in different cell types will now be necessary to validate targets, mediators, and interaction partners, such as 19^ARF^, PRC1 components, and other factors of the DDR, and to explore the function of ROS and ribosomal proteins. Last but not least, the interaction of 2A-DUB/Mysm1 and p53 may not only have an impact on normal development but – similar to the effects of *PcG* genes^47, 65, 66, 67^ – have a role in lymphomagenesis and carcinogenesis.

## Materials and methods

### Mice

2A-DUB/Mysm1^−/−^ mice (Mysm1^tm1Komp/Wtsi^) were obtained from the University of California Davis and were on the C57BL/6N background. p53^−/−^ mice on the C57BL/6 background were a kind gift from Professor KL Rudolph. Unless otherwise indicated, 4- to 6-week-old mice were used for all analyses. All mice were kept in the animal facility of the Tierforschungszentrum of the University of Ulm under specific pathogen-free conditions. All procedures were done in accordance with the guidelines for animal experimentation approved by the Regierungspräsidium Tübingen, Germany.

### Antibodies and FACS analyses

Conjugated antibodies were obtained from either BioLegend Europe Distribution Center (London, UK), eBioscience (Frankfurt, Germany), BD Bioscience (Heidelberg, Germany), or Caltag Laboratories (Darmstadt, Germany), and used in 1 : 200–1 : 400 dilution for FACS analysis. Apoptotic fractions were detected by Annexin V staining (BD Bioscience). Isotype IgG was used as control for all experiments. Cell isolation from mice and subset gating was performed as previously described.^68, 69^ All FACS analyses were performed using a FACS-Canto II System (BD Biosciences) and FACS-Diva, as well as FlowJo Software (Tree Star, Ashland, OR, USA). FACS sorting was performed with a FACS Aria IIu system.

### BrdU labeling and cell cycle analysis

Four-week-old mice were pulsed for 2 h by intraperitoneal injection of 1 mg BrdU per mouse before isolation of BM cells, thymocytes, and splenocytes. Subsequently, cells were stained with surface markers, fixed, and permeabilized, and after DNase incubation, stained with an APC-coupled anti-BrdU antibody using the BrdU Flow Kit (BD Biosciences).

### IF analyses

Fixation, staining, and microscopy of frozen cryosections (5 *μ*m) of thymi from young and aged mice were performed using established protocols as previously described.^68, 70^ TUNEL was performed according to the manufacturer's instructions (*In situ* cell death detection kit, Roche Diagnostics, Mannheim, Germany). To detect apoptotic-related protein expression in thymocyte subpopulations, sections were incubated with Abs against either mouse p19^ARF^ (clone-L3T4, eBioscience), mouse p53 (BD Biosciences), or *γ*H2AX and appropriate secondary Abs. Isotype Ig served as negative control.

### Quantitative PCR

Preparation of total RNA from thymocyte subpopulations, cDNA generation, and qPCR were performed as previously described.^68^ Samples were analyzed in triplicate by qPCR with a LightCycler (Roche Diagnostics), using gene-specific primers for 40 amplification cycles, and expression levels were calculated by normalization of data to GAPDH mRNA expression.

### Chromatin immunoprecipitation

For ChIP assays, proteins were first cross-linked in single-cell suspensions of total thymocytes in 1% formaldehyde, followed by chromatin preparation using the SimpleChIP(R) Enzymatic Chromatin IP Kit (Agarose Beads) and enzymatic chromatin digestion with Micrococcal Nuclease according to the manufacturer's instructions (Cell Signaling, Leiden, The Netherlands). After immunoprecipitation with an antibody against Mysm1 (Santa Cruz Biotech, Heidelberg, Germany), DNA was purified and analyzed by qPCR/PCR in comparison with the input fraction. Subsequently, PCR was performed using the Go Taq Green Master Mix (Promega, Mannheim, Germany).

### BM reconstitution experiments

Rag2^−/−^ CD45.1 congenic mice on the B6.SJL-*Ptprc*^*a*^*Pepc*^*b*^/BoyJ background were kindly provided by Professor HJ Fehling. Twelve lethally irradiated male Rag2^−/−^ SJL mice (2 × 4.5 Gy at an interval of at least 5 h) were reconstituted each with 6 × 10^4^ FACS-sorted Lin-negative BM cells (negative for CD3, CD4, CD8, CD11b, CD19, Gr1, NK1-1, and Ter119) from at least two separate 6- to 8-week-old male wild-type, Mysm1^−/−^ or p53^−/−^Mysm1^−/−^ donors, respectively, via tail vein injection (two recipients per donor). Blood cell counts were analyzed on week 4 after transfer. On week 8 after transfer, recipient mice were killed, and blood and lymphoid organs analyzed for numbers and distribution of T cells and B cells.

### Statistical analysis

Unless otherwise indicated, *P*-values were calculated using Students's *t*-test or one-way ANOVA (analysis of variance) and significance levels denoted as follows: *P<0.05, **P<0.01, ***P<0.005.

## Abbreviations

2A-DUB/Mysm1: Myb-like SWIRM and MPN domain containing1
BM: bone marrow
Cdkn2a: cyclin-dependent kinase inhibitor 2a, Ink4a/Arf
ChIP: chromatin immunoprecipitation
DDR: DNA-damage response
DKO: double knockout
DN: double negative
DP: double positive
DSB: double-strand break
DUB: deubiquitinase
ETP: early thymocyte progenitor
H2A: core histone 2A
HSC: hematopoietic stem cell
LMPP: lymphoid-primed multipotent progenitor
LN: lymph node
PcG: Polycomb group
PRC1/2: Polycomb Repressive Complex 1/2
ROS: reactive oxygen species
SP: single positive
TCR: T-cell receptor
TF: transcription factor
