# Supplementary material for: Interplay of H2A deubiquitinase 2A-DUB/Mysm1 and the p19ARF/p53 axis in hematopoiesis, early T-cell development and tissue differentiation
Source: Cell Death Differ. 2015 Jan 23;22(9):1451–62. doi: 10.1038/cdd.2014.231 (PMC4532772; doi:10.1038/cdd.2014.231)
Supplement: Supplementary Information [file cdd2014231x1.doc]

**Supplemental Materials**

**Figure S1**.

**
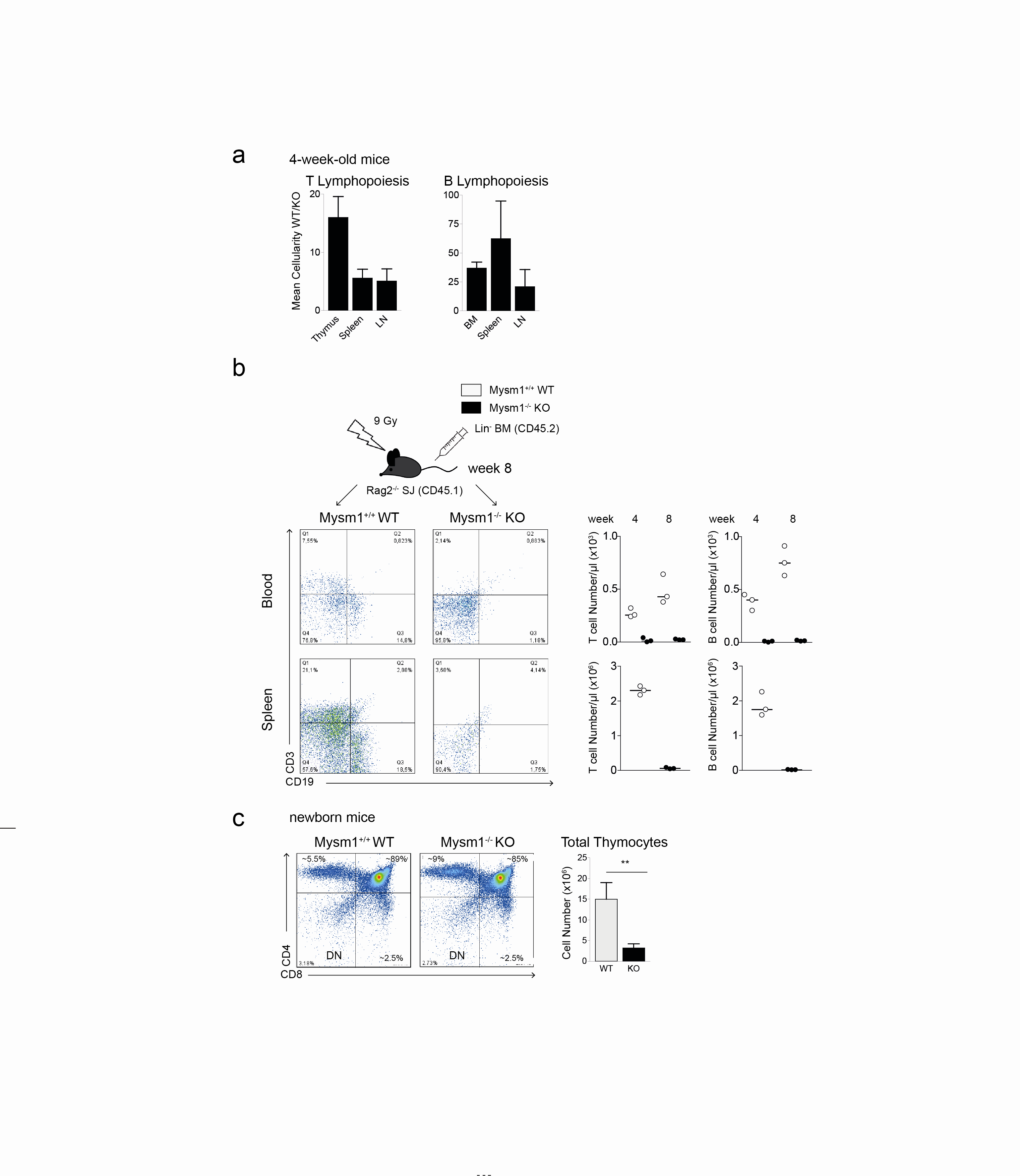
**

**Suppl. Fig. 1**. Defective T cell and B cell development in young and newborn Mysm1^-/-^ mice.

**a**. Overall T cell and B cell numbers were significantly decreased in primary and secondary lymphoid organs of 4-weeks-old Mysm1^-/-^ mice compared with wild-type littermates. Bar graphs show mean ratios of absolute T cell (CD3^+^ cells, left panel) or B cell numbers (CD19^+^ cells, right panel) of wild-type mice versus age-matched Mysm1^-/-^ KO mice in indicated organs. Data represent mean + SD from at least 3 mice of each genotype. **b**. BM reconstitution: FACS-sorted Lin-negative BM cells from Mysm1^-/-^ (KO) or WT littermates were injected into lethally irradiated Rag2^-/-^ SJL recipients. T cell and B cells numbers on week 4 (blood) and week 8 as well as representative FACS plots from individual recipients on week 8 (blood and spleen) after reconstitution with Mysm1^-/-^ KO or WT donor cells are shown (bold lines represent medians, data from 2 independent experiments are pooled). **c**. Reduced thymic T cell numbers in 2-days-old Mysm1^-/-^ mice compared with wild-type littermates. FACS plots (left panel) show representative examples and bar graphs (left panel) represent mean values + SD from 3 independent experiments.

**Figure S2**.


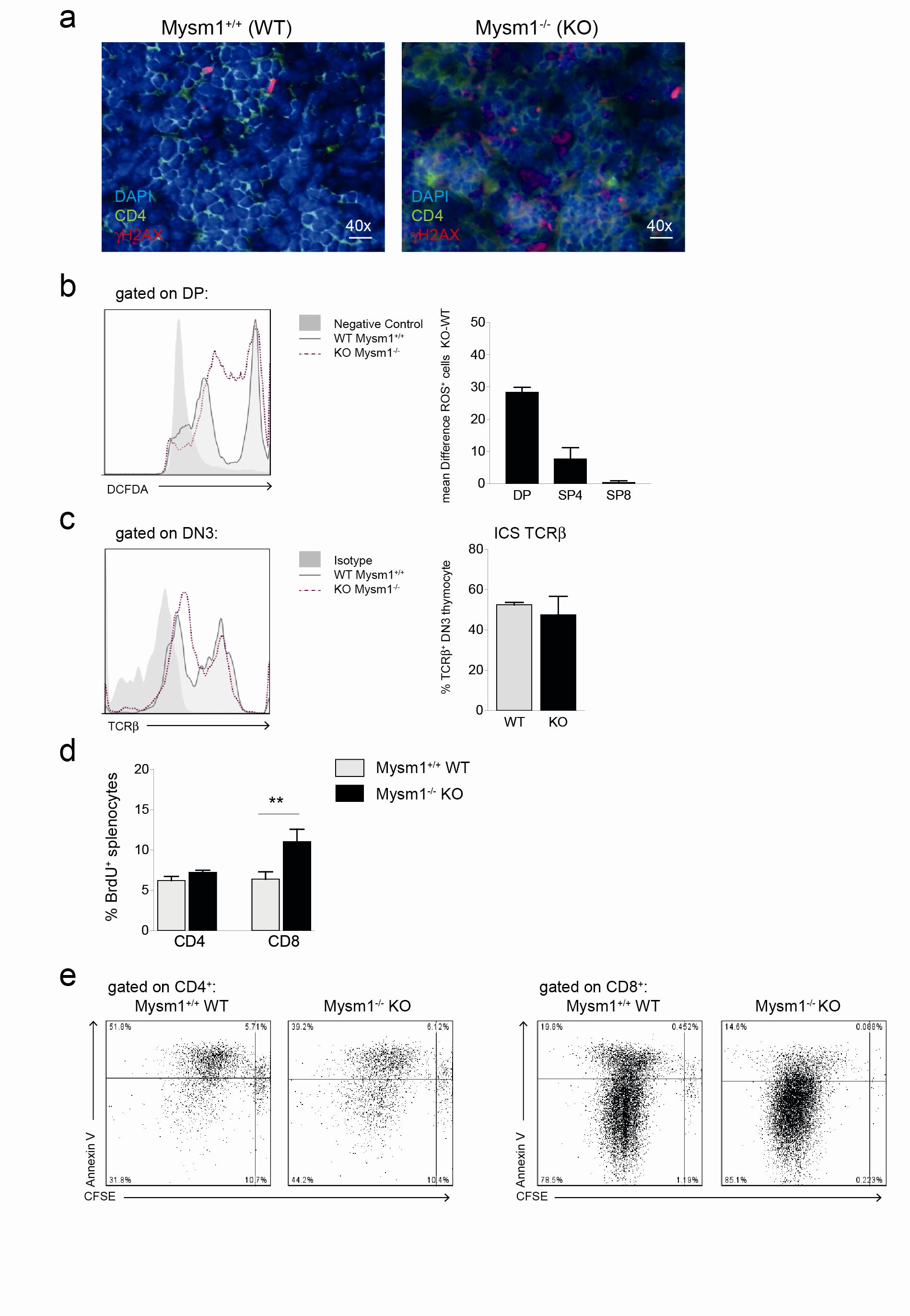


**Suppl. Fig. 2**. Analysis of mechanisms and consequences of defective Mysm1^-/-^ thymic development.

**a**. γH2AX foci in frozen tissue sections of age-matched Mysm1^-/-^ (KO) and Mysm1^+/+^ (WT) thymi (nuclei blue, CD4 green, γH2AX red, white bar corresponds to 10 µm; n=4, representative photographs shown). **b**. Increased apoptosis of Mysm1^-/-^ DP thymocytes correlated with increased ROS levels in comparison with wild-type controls as measured by DCFDA-staining and FACS-analysis. Histograms show representative Mysm1^-/-^ (red dotted line) and wild-type (grey line) stainings gated on DP thymocytes compared to NAC-treated negative controls (grey filled area). Bar graphs represent the difference in the mean percentage of ROS-positive Mysm1^-/-^ versus wild-type thymocytes of indicated subsets + SD of 3 individual experiments. **c**. Intracellular staining for TCRß chain in Mysm1^-/-^ (red dotted line) and wild-type (grey line) DN3 thymocytes in comparison to isotype control antibody (grey filled area). Data in the histogram (left panel) are representative of 3 individual experiments and corresponding bar graphs (right panel) shows mean percentages of TCRß^+^ DN3 thymocytes + SD. **d**. Homeostatic proliferation of peripheral Mysm1^-/-^ or wild-type CD4^+^ and CD8^+^ T cells after a 2 hrs pulse with BrDU. **e**. Proliferation and apoptosis of FACS-sorted Mysm1^-/-^ and wild-type CD3^+^ peripheral T cells in response to plate-bound anti-CD3. CFSE-labelled CD3^+^ T cells (purity > 95%) were seeded at a density of 1x10^6^ cells/ml

in 96-well plated coated with anti-CD3 (final concentration 1 µg/ml) and stimulated for 4 days in the presence of 1 µg/ml soluble anti-CD28 prior to staining with CD4, CD8 and Annexin V for FACS analysis. Representative FACS plots of 2 individual experiments with 4

mice of each genotype shown.

**Figure S3**.


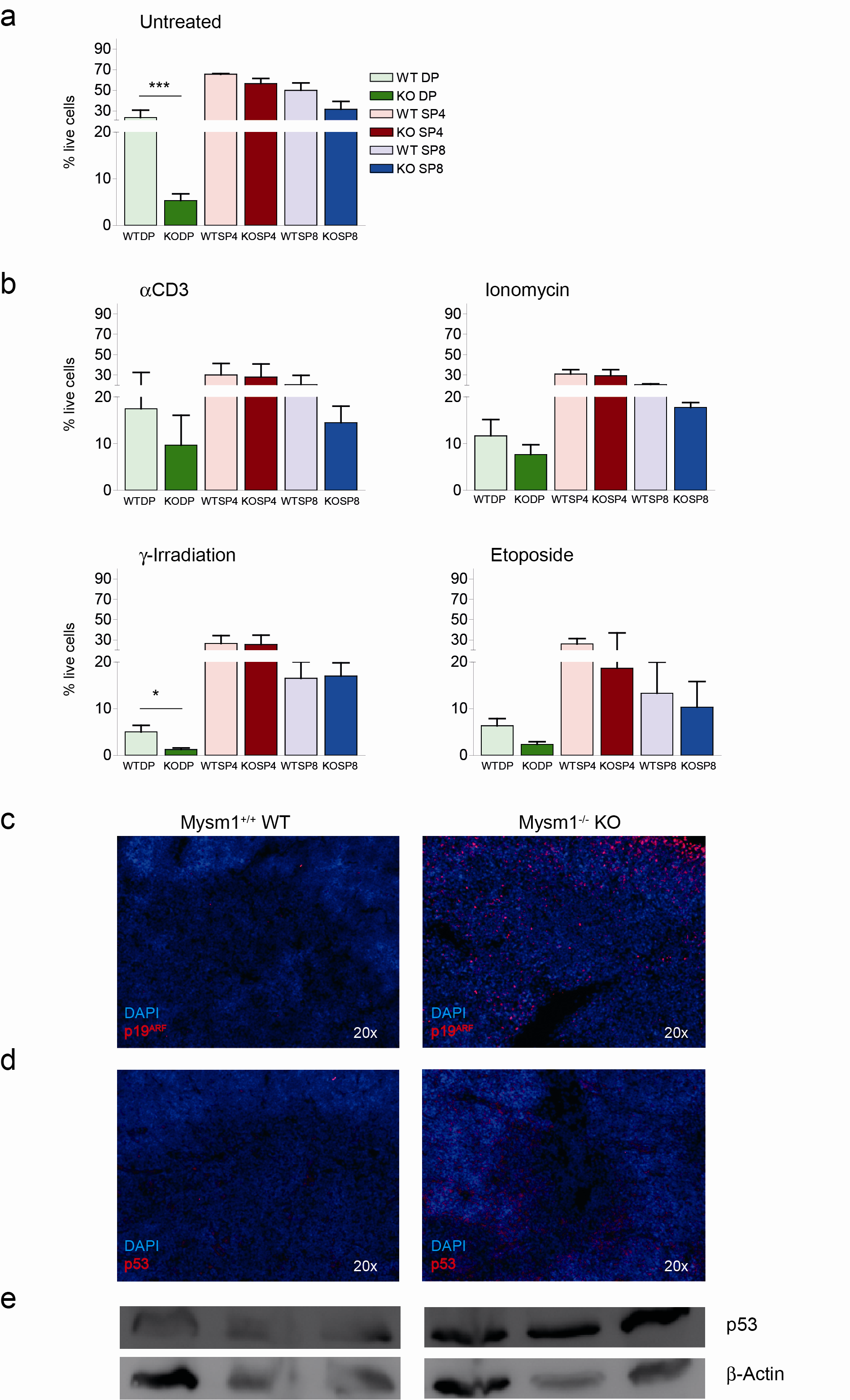


**Suppl. Fig. 3**. Increased apoptosis of Mysm1^-/-^ DP, but not SP, thymocytes in response to starvation, TCR-agonists and DNA-damage.

**a**. Total thymocytes were isolated from 4-weeks-old Mysm1^-/-^ or wildtype mice and either left untreated or **b**. cultured under the indicated conditions for 24 hrs prior to Annexin V-staining to determine apoptotic cell fractions. Bar graphs show the mean percentages of live cells of indicated thymocyte subsets after the 24 hrs culture period + SD (untreated, 1 µg/ml anti- CD3, 1 µM ionomycin, 10 µmol etoposide or gamma-irradiation with 4 Gray). **c**. Immunofluorescence (IF) staining of frozen sections of Mysm1^-/-^ (KO) and wildtype (WT) thymi of 4-weeks old mice with an antibody against p19^ARF^ (red) and nuclear counterstaining with DAPI (blue). **d**. IF analyses of sections from corresponding samples as in c. with an antibody against p53 show increased fractions of p53^+^ cells (red) in Mysm1^-/-^ (KO) thymi. Results of IF analyses are representative of four mice of each genotype (scale bars represent 10 µm, original magnification 40x). **e**. Western blot for total p53 protein in skin samples of 3 3 wt (right) and Mysm1^-/-^ mice (left) in comparison to β-actin as loading control.

**Figure S4**.


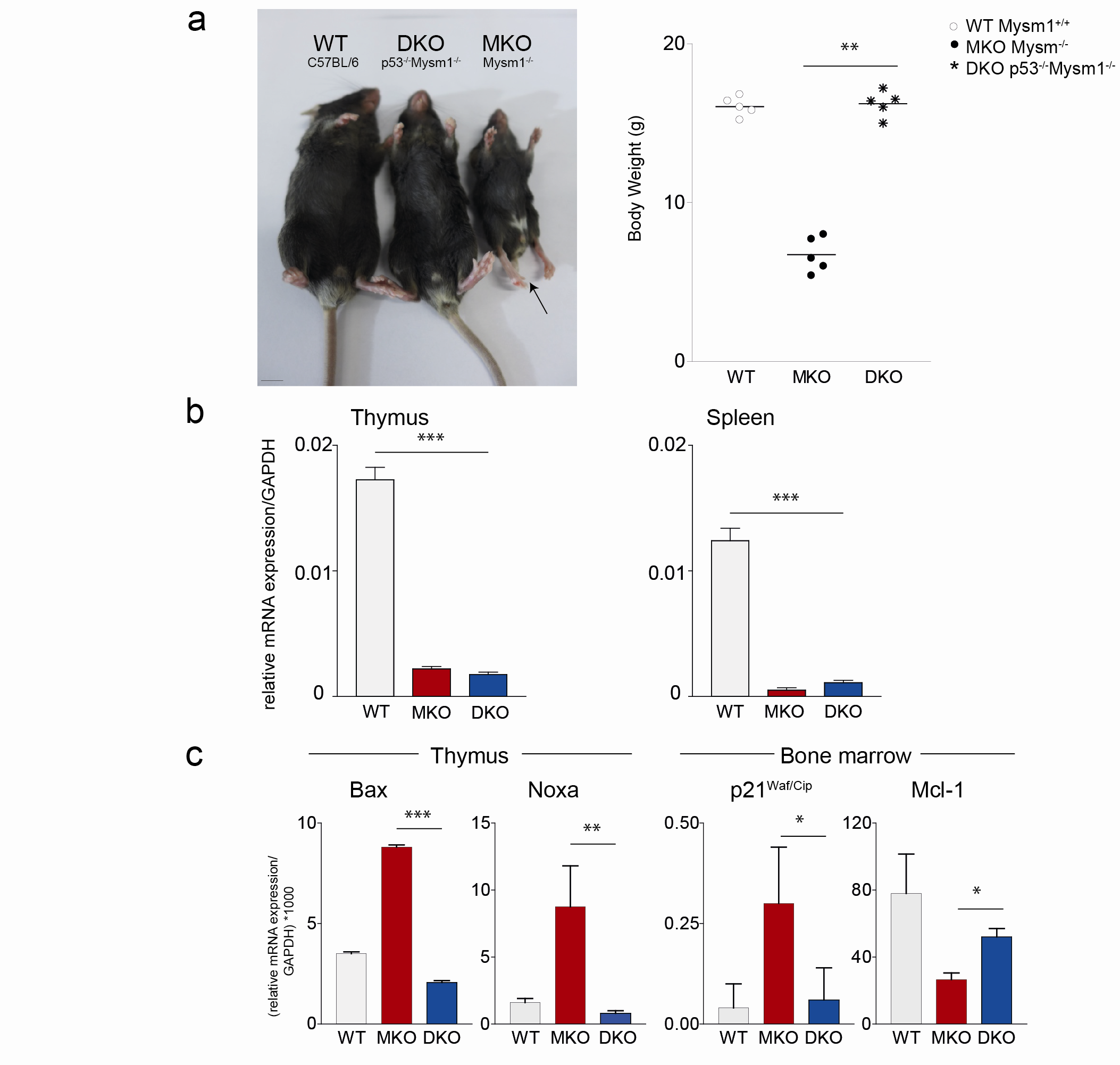


**Suppl. Fig. 4**. General morphology, body weight and Mysm1 mRNA levels of p53^-/-^Mysm1^-/-^DKO mice.

**a**. General morphology and total body weight of 4-weeks-old C57BL/6 wild-type, Mysm1^-/-^ KO and p53^-/-^Mysm1^-/-^ DKO mice. The photo shows representative phenotypes of indicated mice. Scatter plots show the body weight of at least 4 individual mice of each genotype and mean values. **b**. Mean relative Mysm1 mRNA expression + SD of thymi and spleens of at least 3 wild-type, p53^-/-^Mysm1^-/-^ DKO and Mysm1^-/-^ MKO mice relative to GAPDH measured by qPCR. **c**. qPCR analyses showed partial normalization of altered p21^Waf/Cip^, Bax, Puma, Noxa and Mcl-1 mRNA relative to GAPDH in p53^-/-^Mysm1^-/-^ compared with levels in wild-type and Mysm1^-/-^ bone marrow and thymocytes as indicated.
